# Dual regulation of activity and intracellular localization of the PASTA kinase PrkC during *Bacillus subtilis* growth

**DOI:** 10.1038/s41598-018-20145-2

**Published:** 2018-01-26

**Authors:** Frédérique Pompeo, Deborah Byrne, Dominique Mengin-Lecreulx, Anne Galinier

**Affiliations:** 1 Laboratoire de Chimie Bactérienne, UMR 7283, IMM, CNRS, Aix Marseille Univ, 31 Chemin Joseph Aiguier, 13009 Marseille, France; 2 Protein Expression Facility, IMM, CNRS, Aix Marseille Univ, 31 Chemin Joseph Aiguier, 13009 Marseille, France; 3 Institute for Integrative Biology of the Cell (I2BC), CEA, CNRS, Univ Paris-Sud and Université Paris-Saclay, 91198 Gif-sur-Yvette, France

## Abstract

The activity of the PrkC protein kinase is regulated in a sophisticated manner in *Bacillus subtilis* cells. In spores, in the presence of muropeptides, PrkC stimulates dormancy exit. The extracellular region containing PASTA domains binds peptidoglycan fragments to probably enhance the intracellular kinase activity. During exponential growth, the cell division protein GpsB interacts with the intracellular domain of PrkC to stimulate its activity. In this paper, we have reinvestigated the regulation of PrkC during exponential and stationary phases. We observed that, during exponential growth, neither its septal localization nor its activity are influenced by the addition of peptidoglycan fragments or by the deletion of one or all PASTA domains. However, Dynamic Light Scattering experiments suggest that peptidoglycan fragments bind specifically to PrkC and induce its oligomerization. In addition, during stationary phase, PrkC appeared evenly distributed in the cell wall and the deletion of one or all PASTA domains led to a non-activated kinase. We conclude that PrkC activation is not as straightforward as previously suggested and that regulation of its kinase activity via the PASTA domains and peptidoglycan fragments binding occurs when PrkC is not concentrated to the bacterial septum, but all over the cell wall in non-dividing bacillus cells.

## Introduction

Many bacteria possess a conserved family of serine/threonine protein kinases (STPK)^1^ that are involved in the regulation of several cellular processes^2–5^. These enzymes are composed of an intracellular kinase domain resembling the catalytic domain of Hanks-type kinases and, for most, a short transmembrane trait (TM) and an extracellular regulatory C-terminal region containing β-lactam-binding domains^6–8^. These PASTA domains (for penicillin-binding protein and serine/threonine kinase associated domains) are specifically found in bacteria and notably in Firmicutes and in Actinobacteria. They are shown to interact with β-lactam antibiotics^9,10^, peptidoglycan (PG) fragments^6,11^ and Lipid II^12^. Despite a poor conservation of their amino acid sequence, the PASTA domains share a globular fold formed by one α helix facing three β strands^13^. The number of these PASTA repeats varies also among STPKs^1,14^ but they are predicted to be signal-binding domains sensing the state of the cell wall. One of the best-characterized STPKs is the PrkC protein from *B. subtilis*. It is constituted of an intracellular kinase domain, a short transmembrane helix and an extracellular region with a linear modular structure composed of three PASTA domains and a C-terminal domain, which structurally resembles an Ig fold presenting the typical features of adhesive proteins involved in cell-cell interactions or signaling^15,16^. Furthermore, unlike its homologues from other species like *Mycobacterium tuberculosis* or *Streptococcus pneumoniae*, the inactivation of PrkC does not impact cell division, cell shape or cell growth but rather alters stationary phase physiology and spore germination^17–19^. Like its *S. pneumoniae* homologue, PrkC is also expressed during exponential growth; it is localized at the septum of dividing cells and its activity is directly stimulated by the cytoplasmic cell division protein GpsB^20^. During stationary phase growth, *B. subtilis* PrkC seems to be required to limit cell lysis and thus, among the pleiotropic effects of *prkC* deletion, the lysis phenotype would rather be linked to the absence of the elongation factor G (EF-G) phosphorylation^17^. However, in the spore-forming bacteria *B. subtilis*, it has been proposed that the main function of PrkC is to mediate the exit of dormancy in response to PG fragments. Indeed, muropeptide fragments of the cell wall have been shown to stimulate germination of wild-type *B. subtilis* dormant spores whereas they have no effect on *prkC*-mutant spores. This stimulation requires the kinase activity of PrkC since germination is not activated in catalytic mutant (PrkC-K40A) spores^19^. Furthermore, PASTA domains of PrkC were found to bind PG *in vitro* with a clear preference for DAP-type to Lys-type muropeptides^11^ driven by the Arg500 located in PASTA3. The most obvious hypothesis for the muropeptide-driven activation of PrkC would be that their binding to PASTA domains induces dimerization of the extracellular domain thus leading to the formation of the asymmetric active PrkC dimer^16^. Recently, the X-ray structure of the intracellular kinase domain of PknB suggests a model in which a structural and functionally asymmetric ‘front-to-front’ association occurs^21^. The activated autophosphorylated kinase domain can then mediate spore germination^19^ by phosphorylating protein substrates like EF-G. To elaborate this model, the authors demonstrated that, in *Bacillus* spore preparation, EF-G phosphorylation is due to PrkC and to the addition of cell-free supernatants^19^. In another study, the same authors examined EF-G phosphorylation from cells grown to the exponential phase in the presence of PG fragments^22^. Surprisingly, they concluded that PrkC phosphorylates EF-G in response to PG fragments despite the presence of a band corresponding to muropeptide-dependent phosphorylation of EF-G in a Δ*prkC* mutant. This PrkC activation by muropeptides is challenged by other observations. In particular, the catalytic domain alone, without any PASTA motifs, is active *in vitro*^17,23^. Furthermore, a study of the oligomerization state of the extracellular domain of PrkC from *Staphylococcus aureus*, which strongly resembles that of *B. subtilis*, showed that it is monomeric and that muropeptides do not induce its dimerization^16^. The authors suggested that this dimerization mechanism may require the contribution of the TM domain and maybe indirectly modulated by muropeptides. In addition, whereas the PASTA module is required for the autophosphorylation of the kinase IreK from *Enterococcus faecalis* in response to cell wall stress, no direct muropeptide-induced activation of PrkC kinase activity has been observed^24^. In some cases, like for PknB from *M. tuberculosis* and PBP2x or StkP from *S. pneumoniae*, PASTA domains have been shown to be important for cell division and localization of the protein at mid-cell and at the poles^2,25^. In addition, it was recently shown for StkP from *S. pneumoniae* that the PASTA repeats are required for the activation of the kinase independently of muropeptides binding and also to control the septal cell wall thickness^26^. These observations suggest that PASTA domains could have several functions in proteins and are not limited to ligand-binding domains. Thus, the mode of activation of STPKs could be species-specific and more complex than the accepted model described so far.

In this paper, we show that contrary to what was observed for the two homologues PknB or StkP, the septal localization of PrkC is independent of its PASTA domains but depends only on its TM domain in *B. subtilis* growing cells. Addition of PG fragments has no effect on this localization. Furthermore, we demonstrate that the deletion of one or all PASTA domains has no effect on PrkC activity *in vitro* or *in vivo* during exponential growth. In agreement with these data, whereas the protein is able to bind muropeptides, the kinase activity of PrkC is not stimulated by the addition of these PG fragments neither *in vitro* nor *in vivo*. Nevertheless, we show that the PASTA domains are necessary to stimulate PrkC autophosphorylation during the stationary growth phase, when PrkC is no longer concentrated at the septum of the cells but distributed all over the cell wall. All these results suggest that a complex mode of activation for the PrkC kinase activity exists in *B. subtilis* that depends on its cellular localization and is interconnected with bacterial growth phase.

## Results and Discussion

### PASTA domains are not necessary for septal localization of PrkC

**Figure 1:**
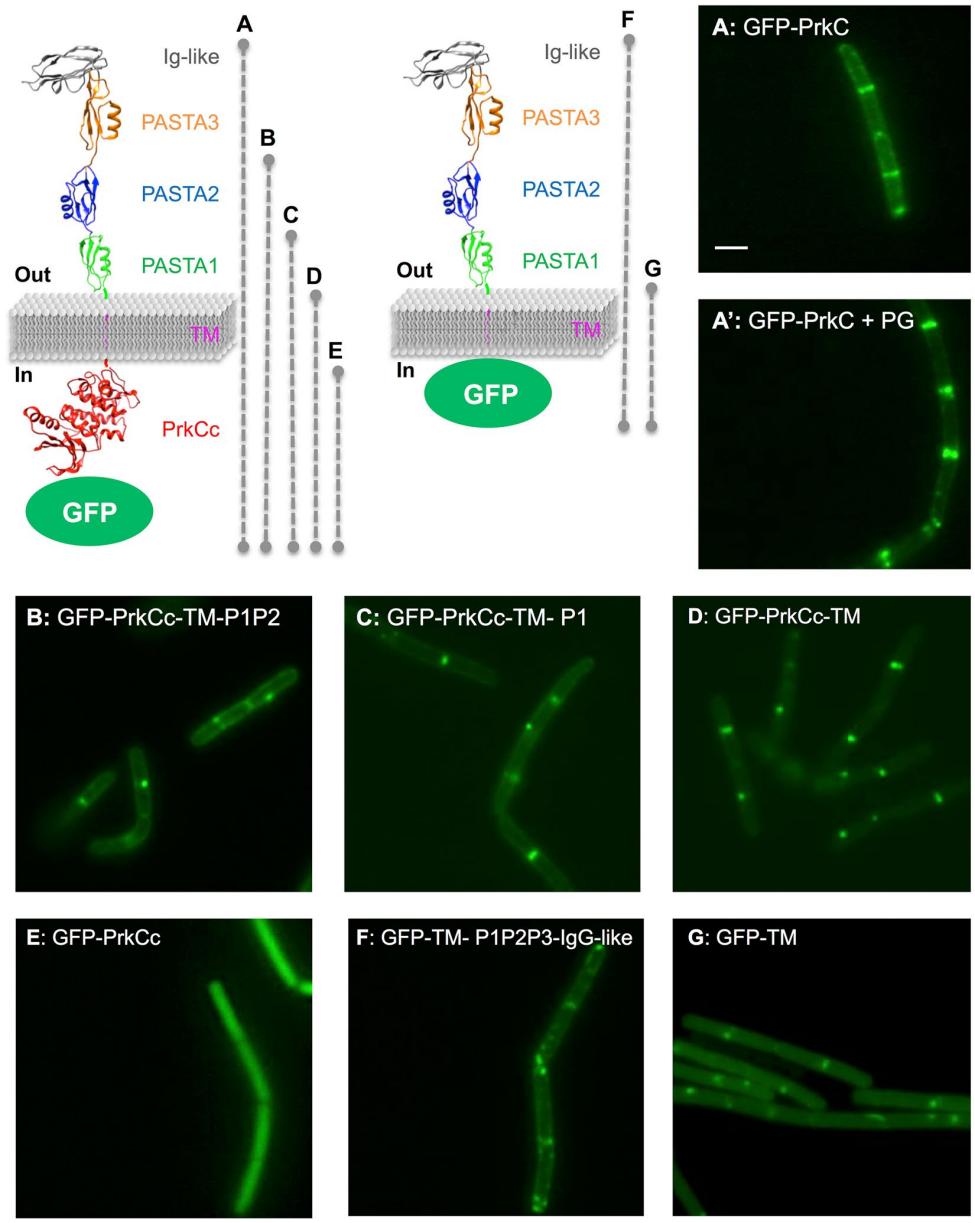
Localization of full-length and truncated GFP-PrkC in *B. subtilis*. 3D representation of GFP-PrkC fusion proteins, with A to G letters and dashed lines indicating the length of the protein. PrkC molecular graphic was modeled with the UCSF Chimera package (supported by NIGMS P41-GM103311) from the 3PY9 PDB structure for the extracellular domain and 4EQM PDB structure for the intracellular domain. Strains were grown on LB medium at 37 °C and all the forms of GFP-PrkC proteins were expressed from the *P*_*xyl*_ promoter in the presence of 0.5% xylose and with 3 µg/ml of PG fragments for the full-length protein. PrkC localization was analyzed by fluorescent microscopy for strains: A: SG278 (Δ*prkC*, *amyE::gfp-prkC*), A’: SG278 in the presence of PG fragments, B: SG467 (Δ*prkC*, *amyE::gfp-prkCc-TM-P1P2*), C: SG466 (Δ*prkC*, *amyE::gfp-prkCc-TM-P1*), D: SG465 (Δ*prkC*, *amyE::gfp-prkCc-TM*), E: SG355 (Δ*prkC*, *amyE::gfp-prkCc*), F: SG497 (Δ*prkC*, *amyE::gfp-TM-P1P2-Ig-like*) and G: SG498 (Δ*prkC*, *amyE::gfp-TM*). The scale bar for microscopy images represents 2 µm.

The extracellular domain has been shown to be required for PknB proper localization at mid-cell and at the poles in *M. tuberculosis*^25^, and for StkP localization at the septum in *S. pneumoniae*^2,27^. To test if the PASTA repeats are also required for PrkC localization at mid-cell and at the poles in *B. subtilis* growing cells^20^, we constructed several PrkC truncated proteins fused to GFP (Fig. 1). These proteins were deleted of one, two or three PASTA repeats as well as other deletions of the cytoplasmic domain or of the entire external domain. We analyzed their localization by fluorescence microscopy and noticed that all the truncated forms of PrkC were properly situated at the septum or at the poles of the cell. The only exception was the catalytic domain alone (GFP-PrkCc) that showed fluorescence throughout the cytoplasm of the bacteria (Fig. 1E). The deletion of one or two PASTA repeats or of all the external domain of PrkC had no effect on its localization (Fig. 1B,C,D). Even the deletion of the catalytic domain, alone or coupled with the deletion of the external domain, had no effect (Fig. 1F and G), as long as the TM domain is present. Since one of the PASTA domain properties is its ability to bind muropeptide fragments of the cell wall, we prepared some PG fragments from an exponential growing cell culture of *B. subtilis* and tested their addition to the cell culture producing GFP-PrkC at the concentration range known to stimulate spore germination as previously described^19^. In agreement with the former observations, this supplement had no effect on the localization of the protein (Fig. 1A and A’). The deletions of PASTA domains or their interaction with PG fragments have therefore no consequences on the localization of PrkC. All these data suggest that its septal and polar localization in *B. subtilis* cells is only mediated by the TM domain. This could be due to the TM domain itself or to its interactions with membrane proteins located at the division sites or at the poles like proteins of the divisome. Some of them may be involved in the localization of PrkC via an interaction of their transmembrane domains. We can already exclude the GpsB, EzrA and DivIVA cell division proteins whose deletion has no effect on PrkC localization^20^. Some proteins recycling the cell wall like hydrolases or PBPs^28,29^ may be potential candidates.

### The deletion of the PASTA domains has no effect on the kinase activity of PrkC *in vitro*

**Figure 2:**
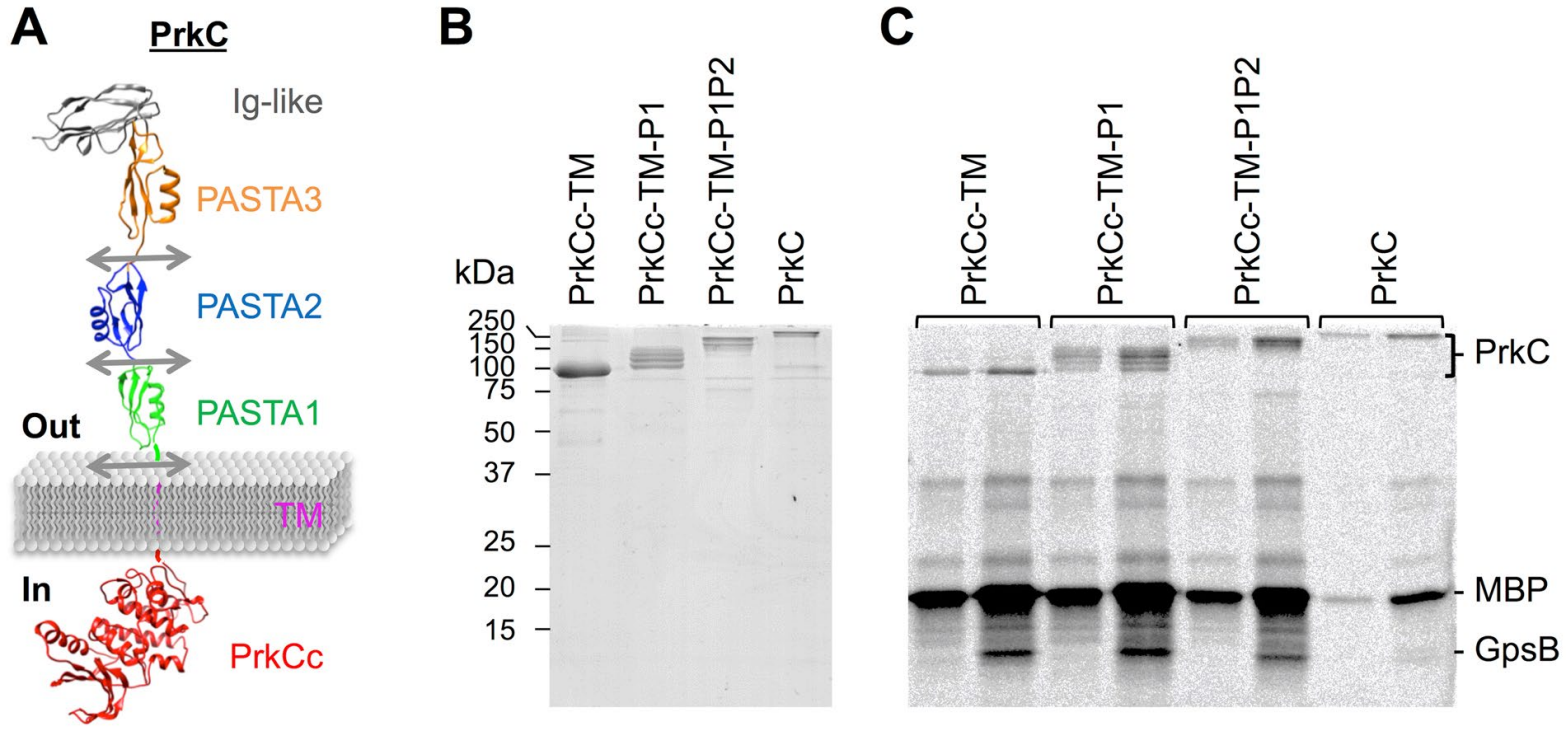
Kinase activity of full-length and truncated PrkC *in vitro*. (**A**) 3D representation of PrkC protein with arrows indicating the different truncation sites. (**B**) Each recombinant PrkC protein was purified and the preparations were visualized on SDS-PAGE gel colored with Coomassie blue. The quantity of purified protein loaded on the gel corresponds to the amount of PrkC protein used in the enzymatic activity test. (**C**) *In vitro* phosphorylation assays in the absence or presence of GpsB for PrkC or the truncated forms of PrkC. The recombinant PrkC proteins were incubated with [γ-^33^P]ATP, MBP and GpsB (lanes 2, 4, 6 and 8) or without GpsB (lanes 1, 3, 5 and 7) at 37 °C during 15 min. The tests were realized in the presence of PrkCc-TM (4.2 µM) (lanes 1 and 2), PrkCc-TM-P1 (2.6 µM) (lanes 3 and 4), PrkCc-TM-P1P2 (1.6 µM) (lanes 5 and 6), or PrkC (0.6 µM) (lanes 7 and 8). Samples were separated by SDS-PAGE and visualized by autoradiography. The radioactive signals seem to decrease with the length of the protein but these differences are due to the lower amounts of protein used in the tests which depend on their concentration after their purification. Full-length gels are presented in the supplemental data.

The catalytic domain of PrkC^30^ alone possesses an efficient kinase activity *in vitro*^17,23,31^. We thus wanted to test if the deletion of the PASTA repeats could have an effect on the enzymatic activity of PrkC *in vitro*. For this purpose, we constructed and then purified some truncated forms of PrkC deleted of one or two PASTAs or of the entire extracellular domain (Fig. 2A). The production and purification yield was low depending on the extracellular region length (Fig. 2B); however, we used these preparations to test the kinase activity of the corresponding proteins *in vitro* with radiolabeled ATP. Since GpsB has been shown to stimulate PrkC^20^, the experiments were also carried out in the presence of GpsB. We observed that the autophosphorylation of PrkC as well as its kinase activity were not significantly modified by the deletions of the PASTA domains (Fig. 2C, odd numbered lanes and Fig. S1). In all cases, the activity was stimulated by GpsB (Fig. 2C, even numbered lanes). These data clearly show that the deletion of any component of the external sensing domain do not significantly affect the kinase activity of PrkC *in vitro* in the absence of muropeptides or other potential enhancer/germinant molecules.

### The binding of muropeptides to PrkC doesn’t modify its kinase activity *in vitro*

**Figure 3:**
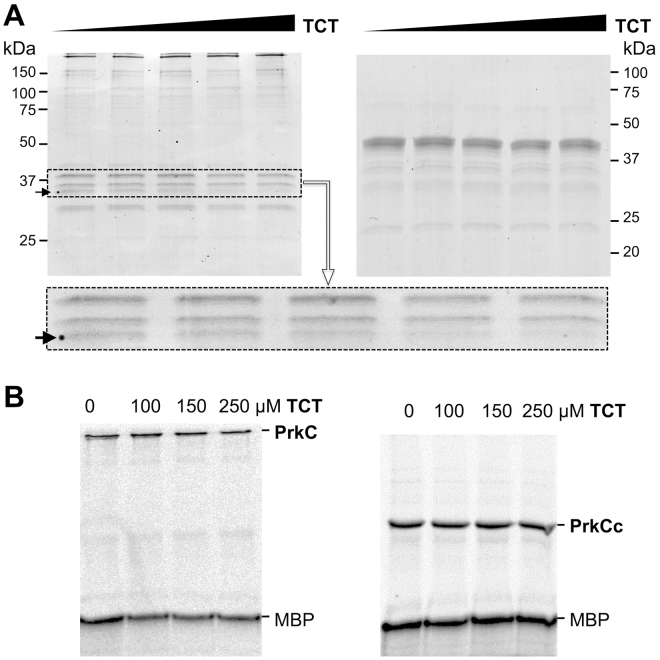
Muropeptides binding to PrkC and its effect on kinase activity *in vitro*. (**A**) Coomassie-stained SDS-PAGE of partial proteolysis profile of the full-length PrkC (left) or its catalytic domain, PrkCc, used as negative control (right). 3 µg of PrkC and PrkCc were incubated with trypsin (Promega) in the absence or presence of increasing amounts of TCT (0, 50, 100, 150 and 250 µM) for 10 min at 37 °C. The digestion profiles were assessed by electrophoresis in SDS-PAGE. For full-length PrkC (left gel), the arrow indicates a band whose intensity decreases in the presence of increasing amounts of TCT. The area where the pattern of digestion is modified by the addition of TCT is framed and magnified under the full-length gel. (**B**) *In vitro* phosphorylation assays in the presence of PrkC or PrkCc. 2.5 µg of protein were incubated with [γ-^33^P]ATP and MBP and increasing amounts of TCT (0, 100, 150 and 250 µM) at 37 °C during 15 min. Samples were separated by SDS-PAGE and visualized by autoradiography. Full-length gels are presented in the supplemental data.

It has been shown by different biochemical approaches that PrkC PASTA repeats and homologues from other species, Stk1, PknB or StkP, are all able to bind muropeptides *in vitro*^10,11,15,19,25,32^. However, these studies were carried out with the extracellular domain alone, in the absence of the catalytic domain, and the effect of this binding on the kinase activity has never been tested so far *in vitro*. In addition, for PrkC from *B. subtilis*, a specific interaction of DAP-type-tetrapeptide was proposed with the PASTA3 by NMR^11^. To test the effect of muropeptide binding on the kinase activity of PrkC, we first checked by limited proteolysis the ability of our purified full-length PrkC protein to bind a purified PG fragments. The analysis of constituent muropeptides of the vegetative cell wall peptidoglycan of *B. subtilis* show that they are mostly composed of glycan strands ending with MurNAc units in the anhydro form and of peptide chains containing three or four amino acids with a clear preference for DAP-amidated-type^33^. We then decided to use the disaccharide tetrapeptide GlcNAc-MurNAc-L-Ala-γ-D-Glu-*meso*-DAP-D-Ala (or TCT)^34^ in our tests (Fig. 3A). As purified muropeptides are usually used at the micromolar range in the previously cited biochemical studies, we decided to test TCT in a scale from 50 to 250 µM. We observed that the digestion profile of PrkC was modified by the addition of TCT suggesting a conformational change due to TCT binding to the protein. Indeed, we observed a decrease in the intensity of a specific protein band (see arrow) with the addition of increasing amounts of TCT (Fig. 3A left gel). This observation could not be made with the PrkCc protein used as negative control (Fig. 3A right gel). These data confirmed that the binding of muropeptides to PrkC protein is dependent of its PASTA domains. This interaction was shown here for the first time by analyzing the entire protein and not only its extracellular domain. The amount of TCT necessary to visualize its binding to PrkC is in agreement with an affinity at the micromolar range for this muropeptide. Furthermore, we tested the effect of this binding on the kinase activity of the same preparation of PrkC using the same amounts of TCT. The catalytic domain PrkCc that is unable to bind the muropeptide was used as negative control. As shown in the autoradiogram, the binding of TCT to PrkC has no effect on its kinase activity *in vitro* (Fig. 3B). These observations were made *in vitro* but clearly run counter to the current activation model of PrkC and we decided to test it *in vivo*.

### Addition of PG fragments to full-length PrkC induces its oligomerization

**Figure 4:**
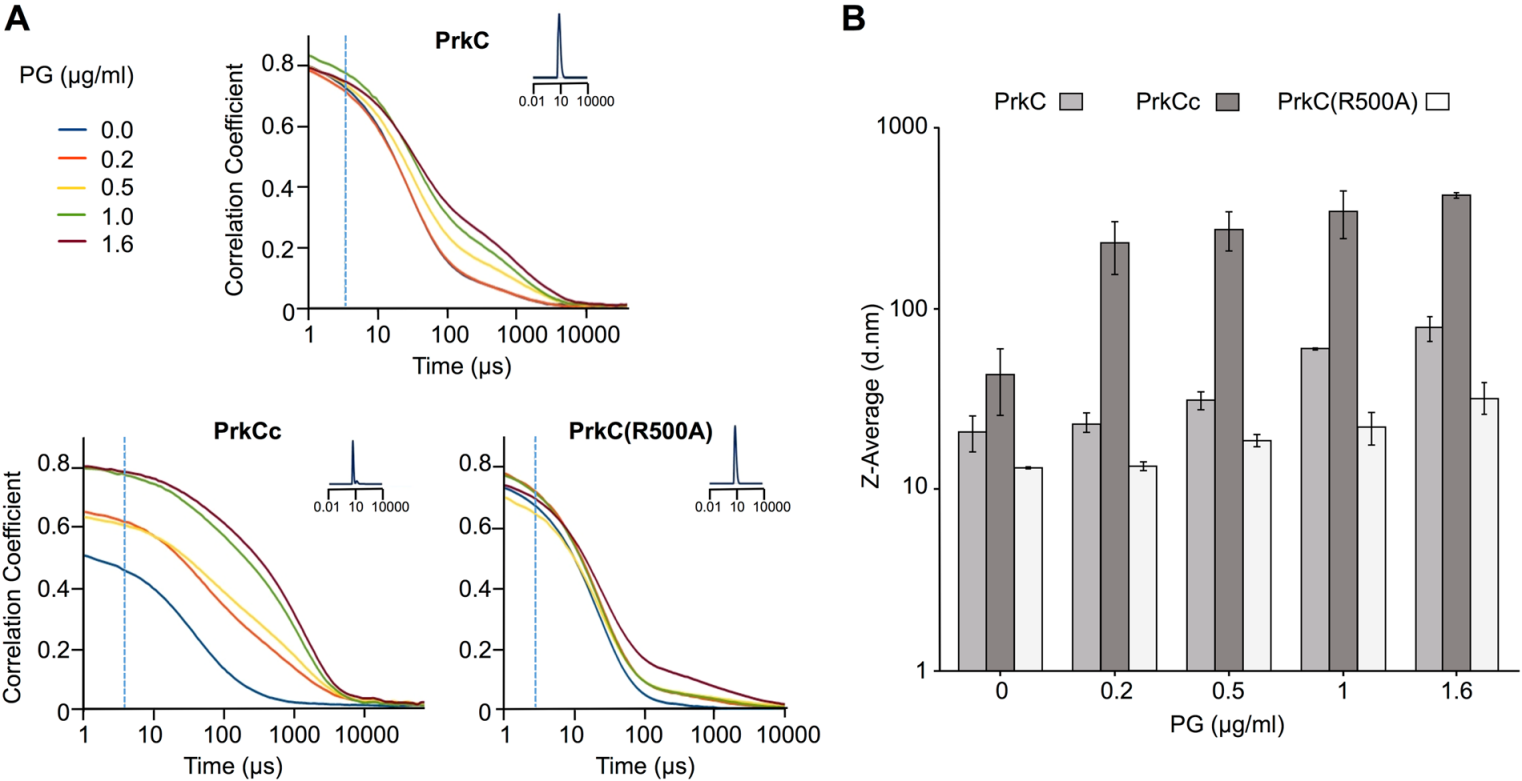
PrkC binds PG. Oligomerization of PrkC, PrkC(R500A) and PrkCc in the presence of increasing concentrations of PG fragments. (**A**) Correlograms displaying raw data of time dependent diffusion of 3 µM PrkC proteins analyzed with increasing concentrations of PG fragments from 0 to 1.6 µg/ml. The insets represent the size distribution by size of PrkC, PrkC(R500A) or PrkCc delta PG mean size. For PrkC and PrkC(R500A), the values of 8.23 and 8.6 hydrodynamic diameters respectively suggests elongated monomers and for PrkCc the value of 4.96 hydrodynamic diameter suggests a globular monomer. Broken blue lines indicate the start of the exponential decay without PG fragments. (**B**) Histogram of PrkC (light grey), PrkCc (medium grey) and PrkC(R500A) (white) Z-average values (mean intensity size) in the presence of increasing concentrations of PG fragments from 0 to 1.6 µg/ml. Z-average gradually increases suggesting an oligomerization effect of PG fragments on PrkC but a lower effect on oligomerization of PrkC(R500A) and infers a non-specific aggregation of PrkCc with PG. Z-average displayed on a logarithmic scale.

The current activation model of PrkC is based on the observation that *B. subtilis* spores of a *prkC* mutant are unable to germinate in response to PG fragments compared to a WT strain^19^, combined to biochemical data showing the interaction between the PASTA3 domain and muropeptides *in vitro*^11^. However, a clear evidence of a direct muropeptide activation of PrkC *in vivo* is still missing. We therefore decided to measure the autophosphorylation of PrkC full-length or of truncated forms of PrkC *in vivo* from cells cultivated in the absence and then in the presence of increasing concentrations of PG fragments. Being limited in the amount of TCT available, we decided to use PG fragments prepared from a *B. subtilis* cell culture stopped at the exponential growth phase and at the concentration range known to stimulate spore germination as previously described^19^. In order to make sure that our PG preparation was containing enough muropeptides able to interact with PrkC, we checked this binding by Dynamic Light Scattering (DLS). We also included the mutant protein PrkC(R500A) as a negative control in the experiment since this mutation, located in the PASTA3, was shown to abolish PG fragments binding. The effect of the increasing amount of PG fragments on the hydrodynamic size of PrkC, PrkC(R500A) and PrkCc respectively was determined by measuring the intensity of the light diffused by the molecules and their translational speed in solution as presented in the correlograms (Fig. 4A). The results showed that PrkC, PrkC(R500A) and PrkCc had hydrodynamic diameters of D_h_ = 8.23 nm, D_h_ = 8.60 nm and D_h_ = 4.96 nm, respectively, by volume distribution median (Dv50) in the absence of PG fragments (Fig. 4A insets) suggesting elongated monomers for PrkC and PrkC(R500A) and a globular monomer for PrkCc. In the presence of 1 µg/ml of PG fragments, PrkC had a D_h_ of 9.20 nm, PrkC(R500A) had a D_h_ of 8.20 nm and PrkCc a D_h_ of 8.40 nm suggesting an oligomerization by PG. However, the analysis of correlograms, Z-averages (Fig. 4B) and diameters by intensity distribution median (Di50) (Fig. S2) suggested a difference between the proteins. All three showed differences in their correlograms in the exponential decay and fluctuating times (Fig. 4A). When PG fragments were added to PrkC, we observed a concentration-dependent increase of the fluctuation time and the broadening of the decay was slow showing a progressive increase in polydispersity suggesting a specific binding of PG fragments to PrkC leading to the oligomerization of the protein. When PG fragments were added to PrkC(R500A), the concentration-dependent increase of the fluctuation time was slower and the broadening of the decay was narrower showing that specific binding of PG fragments to PrkC(R500A) was less efficient and consequently the oligomerization too. Conversely, for the catalytic domain, the addition of PG fragments increased the time at which the correlation begins to decay with a broadening of the fluctuation time suggesting an aggregation of PrkCc by PG fragments and high polydispersity thus a nonspecific binding. These results were confirmed by the Di50 (Fig. S2) and Z-average values (Fig. 4B) that increased constantly and to less than 100 nm with increasing concentrations of PG fragments for PrkC. However, for PrkCc, the Z-average increased beyond 100 nm in the presence of 0.15 µg/ml of PG fragments and continued to increase at 1.5 µg/ml. Small variations of the Z-average values (Fig. 4B) and no variation of Di50 values (Fig. S2) for PrkC(R500A) confirmed that PG fragments binding was impaired on this protein. We also measured the denaturation temperatures for both proteins with or without PG fragments (data not shown). The denaturation temperature of PrkC was identical with or without PG fragments (50 °C) suggesting a good stability of the protein that was not modified by muropeptides binding. On the contrary, the denaturation temperature of PrkCc dropped from 70 °C to 50 °C with the addition of the PG fragments confirming a destabilizing effect of muropeptides on the catalytic domain. Altogether, these results suggest that PG fragments interact specifically with PrkC to induce its oligomerization whereas it interacts non-specifically with PrkCc to induce its aggregation.

### Deletion of PASTA domains or addition of PG fragments has no effect on PrkC activity during exponential phase

**Figure 5:**
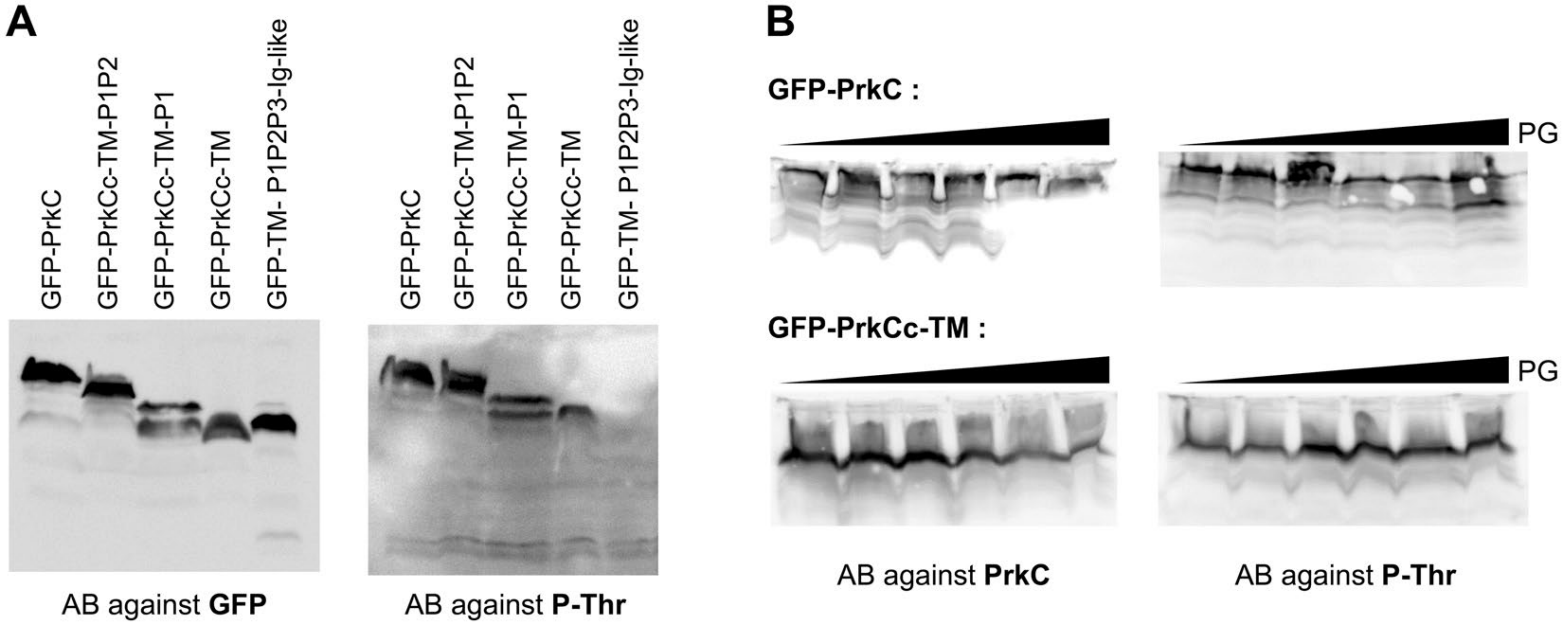
The kinase activity of PrkC *in vivo* is not modified by PASTAs deletions or PG binding during exponential growth. (**A**) PrkC-phosphorylation analysis by western blots. Strains SG278 (Δ*prkC*, *amyE::gfp-prkC*), SG467 (Δ*prkC*, *amyE::gfp-prkCc-TM-P1P2*), SG466 (Δ*prkC*, *amyE::gfp-prkCc-TM-P1*), SG465 (Δ*prkC*, *amyE::gfp-prkCc-TM*) and SG497 (Δ*prkC*, *amyE::gfp-TM-P1P2-Ig-like*) were grown on LB medium with 0.5% xylose at 37 °C until OD_600_ = 0.8. After centrifugation, the pellets were resuspended in 1/100^th^ volume of lysis buffer and treated as described in Materials and Methods. For each strain, 16 μl of crude extract were separated by SDS-PAGE. After blotting, phosphorylated PrkC was detected using antibodies directed against P-Thr residues. To estimate the relative quantity of PrkC in crude extracts, we used anti-GFP antibodies. (**B**) Strains SG278 (Δ*prkC*, *amyE::gfp-prkC*) and SG465 (Δ*prkC*, *amyE::gfp-prkCc-TM*) were grown on LB medium with 0.5% xylose and increasing amounts of PG fragments (0, 0.003, 0.015, 0.03, 0.3 and 3 µg/ml) at 37 °C until OD_600_ = 0.8. After centrifugation, the pellets were resuspended in 1/100^th^ volume of lysis buffer and treated as described in Materials and Methods. For each strain, 16 μl of crude extract were separated by SDS-PAGE. After blotting, phosphorylated PrkC was detected using antibodies directed against P-Thr residues. To estimate the relative quantity of PrkC in crude extracts, we used a specific antibody against PrkC. Full-length blots are presented in the supplemental data.

Since PG fragments bind to PASTA domains, we first analyzed the effect of PASTA deletions on PrkC activity in *B. subtilis* growing cells. For this purpose, strains expressing the truncated-PrkC-GFP proteins were grown in a rich medium until the mid-exponential phase (OD_600_ = 0.8) and crude extracts were prepared and analyzed by western blots. Using antibodies against the GFP protein, we first checked that all the forms of PrkC were produced at the same level in the cell (Fig. 5A). We then measured the autophosphorylation of PrkC *in vivo* using antibodies against phospho-threonine (P-Thr). We observed that all the forms of PrkC were phosphorylated except the extracellular domain of PrkC (GFP-TM-P1P2-Ig-like) lacking the catalytic intracellular kinase domain that served as the negative control. These data clearly show that all the forms of PrkC containing the catalytic domain are active. However, the absence of the PASTA domains has no effect on PrkC autophophorylation and therefore on its kinase activity *in vivo*. This observation has also been made recently for the homologous protein IreK from *E. faecalis* but to a lesser extent^24^. Indeed, a truncated form of IreK lacking its five PASTA repeats was still active indicating that the extracellular domain of IreK is not required for an answer to a signal associated with growth and/or cell division. However, the specific stimulation of IreK kinase activity in response to cell-wall antimicrobials seemed to be dependent of the PASTA module, which suggests multiple parameters for sensory input *in vivo*^24^.

Since our preparation of PG fragments contains muropeptides able to bind to PrkC, we decided to test it on PrkC autophosphorylation *in vivo*. For this, strains producing GFP-PrkC or GFP-PrkCc as negative control were grown in a rich medium in the presence of increasing amount of PG fragments until the mid-exponential phase (OD_600_ = 0.8). Then crude extracts were prepared and analyzed by western blots. Using antibodies against PrkC, we observed that both proteins were produced at the same level whatever the amount of PG fragments added (Fig. 5B). In addition, the phosphorylation signal detected in all lanes with antibodies against P-Thr showed that the level of PrkC autophosphorylation was similar (Fig. 5B) regardless of the form of the protein. This result indicates that PrkC kinase activity is not stimulated *in vivo* by PG binding to the PASTA repeats during exponential growth.

### PASTA domains are necessary for PrkC activation during stationary phase growth

**Figure 6:**
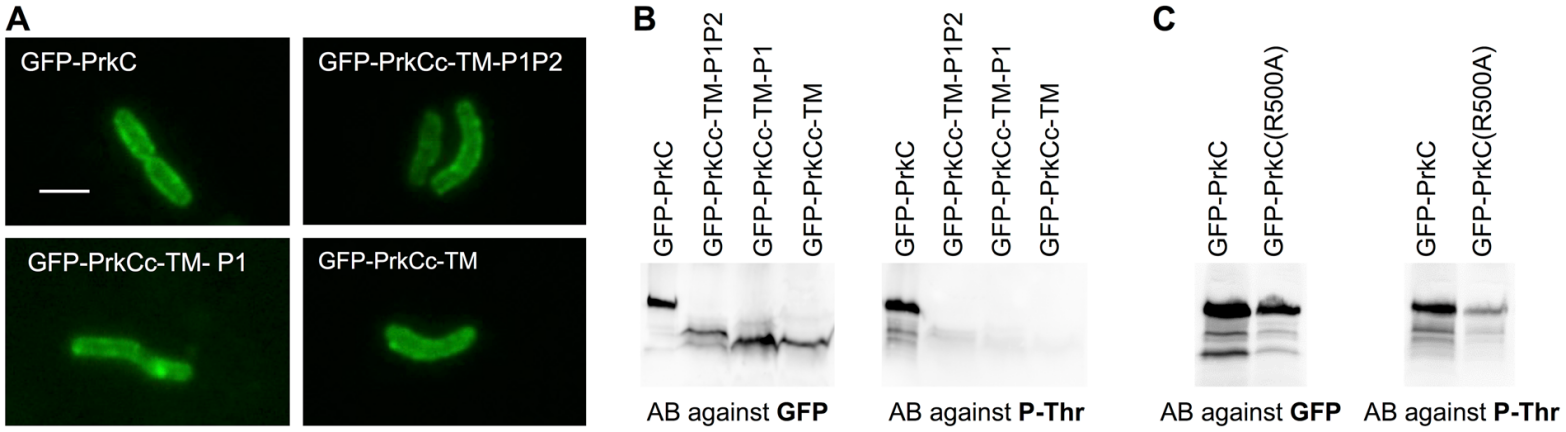
Localization of PrkC during stationary phase growth and role of the PASTA domains. (**A**) PrkC localization during stationary phase growth. Strains were grown on LB medium at 37 °C during 23 hours and all the forms of GFP-PrkC proteins were expressed from the *P*_*xyl*_ promoter in the presence of 0.5% xylose. PrkC localization was analyzed by fluorescent microscopy for strains SG278 (Δ*prkC*, *amyE::gfp-prkC*), SG467 (Δ*prkC*, *amyE::gfp-prkCc-TM-P1P2*), SG466 (Δ*prkC*, *amyE::gfp-prkCc-TM-P1*) and SG465 (Δ*prkC*, *amyE::gfp-prkCc-TM)*. The scale bar for microscopy images represents 2 µm. (**B**) PrkC-phosphorylation analysis by western blots. The same strains SG278, SG467, SG466 and SG465 were grown on LB medium with 0.5% xylose at 37 °C for 23 hours (final OD_600_~ 6). After centrifugation, the pellets were resuspended in 1/30^th^ volume of lysis buffer and treated as described in Materials and Methods. For each strain, 16 μl of crude extract were separated by SDS-PAGE. After blotting, phosphorylated PrkC was detected using antibodies directed against P-Thr residues. To estimate the relative quantity of PrkC in crude extracts, we used anti-GFP antibodies. (**C**) PrkC(R500A)-phosphorylation analysis by western blots. The strains SG278 and SG622 were grown on LB medium with 0.5% xylose at 37 °C for 23 hours (final OD_600_~6) and the culture pellets were treated as mentioned in (**B**). Full-length blots are presented in the supplemental data.

Since we did not detect any effect of PASTAs deletions or addition of PG fragments during exponential phase, we decided to investigate the localization and the activity of the protein in stationary phase cells. We first analyzed the localization by fluorescence microscopy of GFP-PrkC as well as of the truncated forms of PrkC deleted of one or several PASTA repeats. Unexpectedly, we noticed that all the forms of PrkC were distributed all over the cell wall (Fig. 6A). These observations revealed, for the first time, that the localization of PrkC varies according to the growth phase (Fig. S3). However, the PASTA domains are not necessary for this new positioning of the kinase. Thus, PrkC is concentrated at the septum and at the poles during cell division and delocalizes throughout the cell wall when the cells enter into a non-dividing state, but in both cases, the external domain is not required for PrkC cellular localization. We then wanted to test if a role of the PASTA domains would be to stimulate the kinase activity by autophosphorylation in non-dividing cells. We thus prepared crude extracts from stationary phase cultures producing the PASTA-truncated forms of GFP-PrkC or the mutant protein GFP-PrkC(R500A) unable to bind PG fragments and to oligomerize. These crude extracts were then analyzed by western blots using antibodies against the GFP protein to check that all the forms of PrkC were produced at the same level in the cell and using antibodies against P-Thr to measure the autophosphorylation of PrkC *in vivo* (Fig. 6B and C). Whereas all forms of PrkC were expressed at the same level, only the entire protein was still detected by the antibodies against P-Thr. The removal of the PASTA3 or the mutation of the Arg500 to Ala, known to interact with PG fragments, was sufficient to lose the phosphorylation signal. These results clearly show that, during stationary phase growth, the binding of PG fragments to the extracellular domain of PrkC is required to allow the autophosphorylation of the protein and therefore to stimulate its kinase activity.

### *A* sophisticated model for kinase activation of PrkC

**Figure 7:**
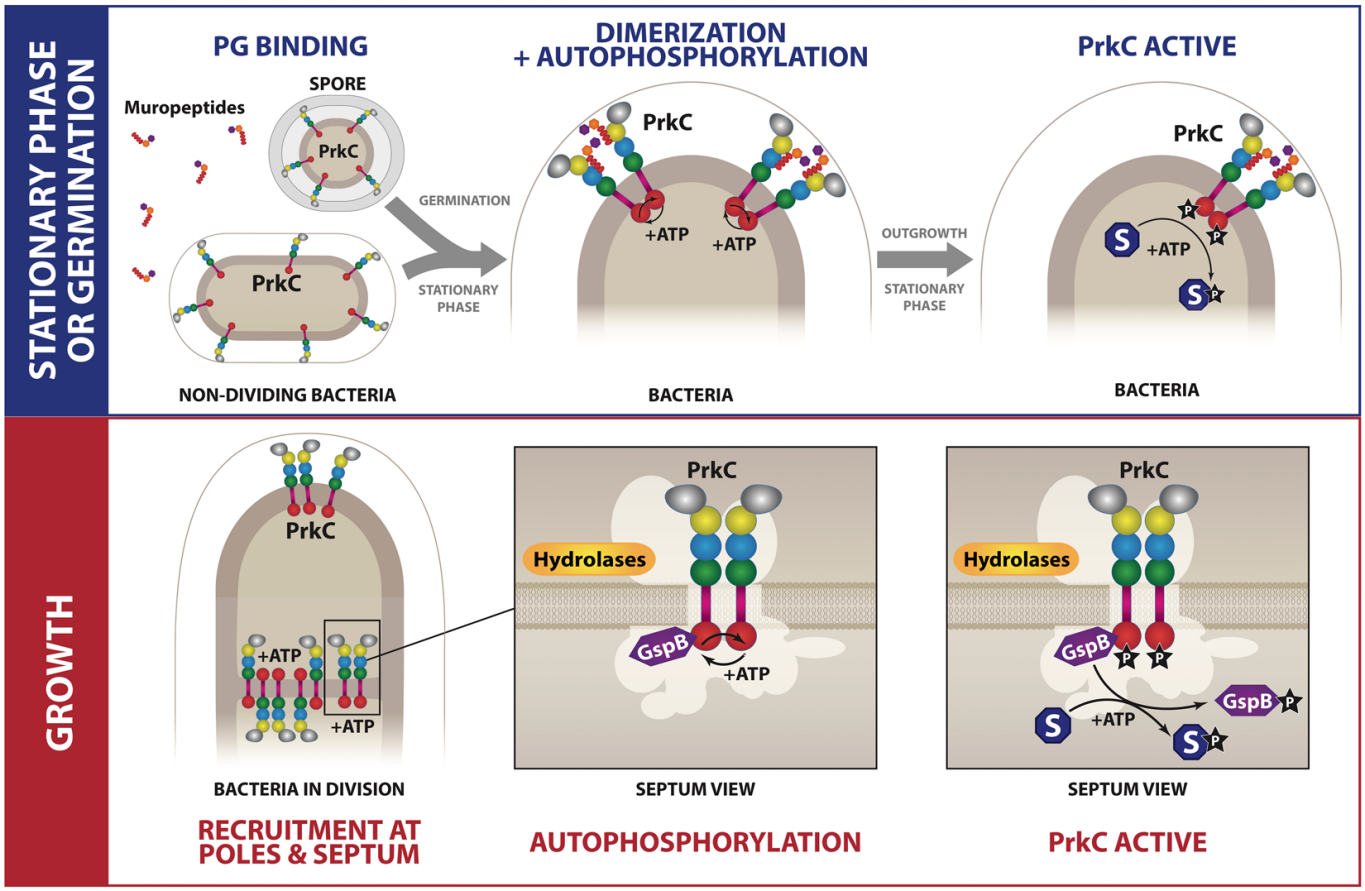
Two activation modes for PrkC. Two activation modes are proposed for PrkC depending on the physiological state of the bacteria. On the top panel (in blue), we designed the model of activation during stationary phase growth or germination of *B. subtilis* spores. During stationary phase or before germination, PrkC is distributed all over the cell wall of the bacteria or in the internal membrane of the spore. The binding of muropeptides (germination signal for spores) stimulates PrkC dimerization and autophosphorylation. The activated PrkC can then phosphorylate its cytoplasmic substrates. On the panel below (in red), we designed the model of PrkC activation during bacterial growth. Thanks to interactions with membrane proteins at the poles and at the septum, like hydrolases or proteins of the divisome schematically represented by the white shadow, PrkC molecules get closer to each other. PrkC interacts also with the division protein GpsB which stimulates its autophosphorylation and its kinase activity. The activated PrkC is then able to phosphorylate its cytoplasmic substrates. The feedback loop regulation of PrkC activity by phosphorylated GpsB protein^20^ is not described here. The divisome schematically represented by the white shadow includes the proteins described in^37^ and containing EzrA, PBP1, PBP2b, ZapA, SepF, FtsZ, FtsA, FtsL, FtsW, DivIB, DivIC, and DivIVA.

Our data suggest that PrkC regulation is a complex mechanism that may vary depending on the physiological state of the cell, i.e. growing cells versus non-dividing cells (stationary phase cells or spores) and according to the ligand and the partners of the protein when it is involved in one cellular process or another. We therefore propose a new model for the modulation of PrkC kinase activity depending on its cellular localization, the growth phase and the cellular process regulated (Fig. 7). The current model described for germination conditions and which can also be proposed for stationary phase conditions is summarized in (Fig. 7 top panel) and the designed regulatory model for PrkC kinase activity during exponential growth is presented in (Fig. 7 lower panel). During stationary phase (or germination), PrkC is located all around the cell wall (or the spore membrane), PG fragments are available, and the cells (or spores) probably need to sense an environmental signal to ensure the best rate of cell survival (or induce the exit from dormancy). Thus, stimulation by PG fragments through the PASTA domains of the protein may be required to induce PrkC dimerization and thus kinase activation via its autophosphorylation. This layout could be similar in other sporulating bacteria in which a PASTA-containing protein kinase is involved in spore germination. During growth, PrkC is concentrated at mid-cell or at the poles and, in such conditions, it likely has no access to the extracellular medium and thus to free PG fragments. Actually, no stimulation of its kinase activity by addition of PG fragments has been detected *in vivo* but it is stimulated by interaction with the division protein GpsB^20^. Moreover, we did not detect any stimulation of the kinase activity by TCT *in vitro*, although it binds to PrkC. This could be explained by the strong probability for two protein molecules to be in contact in solution before the addition of TCT and to phosphorylate each other. A high percentage of the PrkC molecules are therefore already active. *In vivo*, ligands like lipid II or other periplasmic molecules may play a role in PrkC dimerization that can also be mediated by its recruitment, via its TM domain, by partners located at the septum and/or at the poles like proteins of the division machinery, PBPs and hydrolases or other membrane associated proteins. During cell division, PrkC molecules are thus focused at the poles and septa where they can phosphorylate each other to become active. Furthermore, GpsB and to a lesser extent EzrA and DivIVA, increase PrkC kinase activity via a yet unknown mechanism. In these conditions, the stimulation by PG fragments may not be necessary. Our results are consistent with the regulation observed in *E. faecalis* cells, where IreK can respond to cell wall stress by enhancing its kinase activity via its PASTA repeats and can also respond to signal associated with growth and/or division independently of the presence of its extracellular PASTA-containing domain^24^. We can conclude that PASTA-kinases are subtly stimulated according to their cellular localization and the cellular processes they regulate. This fine-tune regulation may also differ between species.

## Methods

### Plasmids and strains constructions

Standard procedures for molecular cloning and cell transformation of *B. subtilis* and *E. coli* were used. All the strains and plasmids used in this study are listed in Table 1. Primers used in this study are available upon request. Sequencing of PCR-derived DNA fragments in the plasmid constructs was carried out by GATC-Biotech to ensure error-free amplification.

**Table 1:** *B. subtilis* strains and plasmids used in this work.

Strain/plasmid Relevant characteristics		Source/reference
***B. subtilis*** **strains**		
WT168	*trpC2*	lab collection
1A963	*trpC2 prkC∆1*	^17^
SG278	*trpC2 prkC∆1, amyE::gfp-prkC*	^20^
SG355	*trpC2 prkC∆1, amyE::gfp-prkCc*	This work
SG465	*trpC2 prkC∆1, amyE::gfp-prkCc-TM*	This work
SG466	*trpC2 prkC∆1, amyE::gfp-prkCc-TM-P1*	This work
SG467	*trpC2 prkC∆1, amyE::gfp-prkCc-TM-P1P2*	This work
SG497	*trpC2 prkC∆1, amyE::gfp-TM-P1P2P3-Ig-like*	This work
SG498	*trpC2 prkC∆1, amyE::gfp-TM*	This work
SG622	*trpC2 prkC∆1, amyE::gfp-prkC(R500A)*	This work
**Plasmids**		
psG1729-*prkCc*		This work
psG1729-*prkCc-TM*		This work
psG1729-*prkCc-TM-P1*		This work
psG1729-*prkCc-TM-P1P2*		This work
psG1729*-TM-P1P2P3-Ig-like*		This work
psG1729*-TM*		This work
psG1729-*prkC(R500A)*		This work
pETDuet-*prkC*		This work
pETDuet-*prkC-TM*		This work
pETDuet-*prkC-TM-P1*		This work
pETDuet-*prkC-TM-P1P2*		This work
pETDuet-*prkC(R500A)*		This work

For the generation of fluorescent fusion proteins, the truncated versions of the *prkC* gene were amplified by PCR using specific primers allowing its insertion between *Apa*I and *Xho*I sites in pSG1729^35^. The *B. subtilis* strain 1A963^17^ was then transformed with pSG1729*-gfp-prkC-(truncated versions)* or pSG1729*-gfp-prkC(R500A)* generating the strains SG355, SG465, SG466, SG467, SG497, SG498 and SG622. Protein expression was induced with 0.5% xylose (w/v).

The truncated or mutated versions of PrkC were overexpressed in *E. coli*, with the corresponding genes amplified by PCR using *B. subtilis* 168 genomic DNA as a template and a primer pair containing *Sac*I and *Pst*I restriction sites. The amplified products were digested with *Sac*I and *Pst*I and then ligated to the pETDuet vector. The resulting plasmids are listed in Table 1.

### PG preparation and purification of TCT muropeptide

*B. subtilis* PG fragments from growing cells were prepared as previously described in^19^. The pellet containing the cell wall PG freed of proteins and lipoteichoic acids was quantified by weighing then resuspended and digested with mutanolysin (10 µg/ml) overnight at 37 °C prior to inactivation of the enzyme at 80 °C for 20 min. The concentration of PG fragments was determined by quantitative aminoacid (diaminopimelic acid) and aminosugar (muramic acid, glucosamine) analysis with a Hitachi L8800 analyzer (ScienceTec, Les Ulis, France) after hydrolysis of samples in 6 M HCl at 95 °C for 16 h. PG fragments were then used in the several tests. TCT muropeptide was produced and purified as described in^36^.

### Dynamic Light Scattering

Dynamic light scattering (DLS) experiments were carried out to determine the hydrodynamic diameter of PrkC, PrkC(R500A) and PrkCc in the presence and absence of PG using a Zetasizer Nano ZS (Malvern Instruments). Particles in solution are in a constant random motion and the intensity of the scattered light fluctuates with time. To determine the particle size, the provided software uses the Stokes-Einstein relation to obtain the intensity averaged size distribution from the raw correlation data. The correlograms give several information about the sample. The time at which the correlation starts to decay is an indication of the mean size of the sample, smaller samples fluctuate quicker than larger samples is solution. The steeper the exponential decay, the more monodisperse (single population) the sample, and if the decay is extended the greater the sample polydispersity (several populations). Size distribution assays were performed at 25 °C. For each assay three measurements were performed; each one consisting in 10–15 runs of 10 seconds. The scattering angle was 173°. Temperature trend assay to calculate aggregation points contained a sequence from 20 to 70 °C with 10 °C intervals. We displayed our results using the correlograms, Z-average (mean intensity size of sample) and Di50 (diameter by intensity of 50% of the molecules in solution) using the Zetaziser software 7.12. PrkC and PrkC(R500A) were prepared at 0.2 mg/ml in 20 mM Tris-HCl pH 7.5, 200 mM NaCl, 7% glycerol following centrifugation for 15 min at 14000 rpm at 6 °C. PrkCc was prepared at 0.1 mg/ml in 20 mM Tris-HCl pH 7.5, 200 mM NaCl, 7% glycerol following centrifugation for 15 min at 14000 rpm at 6 °C. The proteins were analyzed in the absence or presence of increasing concentrations of PG fragments from 0.15–1.6 µg/ml.

### Microscopy

Strains were grown on LB medium at 37 °C. The *gfp-prkC* gene fusion and all truncated versions of *prkC* were expressed from the inducible P_*xyl*_ promoter in the presence of 0.5% xylose. The PrkC localization was analyzed by fluorescent microscopy on a Zeiss Upright Axio Imager M2 microscope as described previously^3^.

### Western Blot

The cells were grown at 37 °C in 30 ml of LB medium to OD_600_ = 0.8 for exponential phase extracts and to OD_600_~6 for stationary phase extracts (23-hour cultures) then centrifuged for 10 min at 8000 rpm at 4 °C. When needed, the exponential phase cultures were realized in the presence of increasing amounts of PG fragments (0, 0.003, 0.015, 0.03, 0.3 and 3 µg/ml). Cell pellets were resuspended in 1/100^th^ and 1/30^th^ volumes of lysis buffer for exponential and stationary phase cultures, respectively, containing 10 mM Tris-HCl pH 8.0, 150 mM NaCl, 0.1% NP40, 1 mM PMSF, 1 mM DTT, 25 U.ml^−1^ benzonase and 10 mg.ml^−1^ lysozyme, and incubated for 30 min at 37 °C. 1/10^th^ volume of 10% SDS and 1/2 volume of (2×) Laemmli buffer were added to the extracts that were heated at 100 °C for 10 min. Samples were run on a 10% or 12.5% SDS-PAGE and transferred to hybond-ECL membrane by electroblotting. The membrane was blocked with PBS solution containing 5% milk powder (w/v), for 3 hours at room temperature with shaking, then incubated with anti-GFP or anti-PrkC antibodies diluted to 1/10000^th^ or 1/1000^th^, respectively, overnight at 4 °C. After three washes, the secondary antibody, a peroxidase-conjugated Goat anti-Rabbit (DAKO) antibody, was used at 1/10000^th^ dilution for one hour. After three washes, the membrane was incubated with ECL reagents (Perkinelmer) and scanned for chimioluminescence with an ImageQuant LAS4000 (GE Healthcare). A second membrane was used for anti-P-Thr antibodies as previously described in^20^.

### Protein purification

Plasmids overproducing 6His-tagged PrkC truncated proteins were used to transform *E. coli* C41(DE3). Purification of 6His-tagged recombinant proteins was performed with Ni^2+^-NTA resin (Qiagen) as previously described in^18^ for PrkCc, the PASTA-truncated forms of PrkC, PrkC and PrkC(R500A). Before purification, in order to solubilize the proteins containing a TM domain, 0.4% Triton X100 was added to the crude extracts, shacked for 1 h at 4 °C then centrifuged at 35000 rpm for 1 h at 4 °C to removed membrane fragments. In addition, the buffer used for the purification steps of these proteins contained 0.2% Triton X100.

### Protein phosphorylation

2 μg of GpsB were incubated for 15 min at 37 °C with 2.5 μg of the several forms of PrkC protein in a 15 μl reaction mixture containing 10 mM HEPES, pH 8.0, 1 mM MgCl_2_, 2 μg of MBP (Myelin Basic Protein) that was shown to be phosphorylated by PrkC^18^ and 1 mM [γ-^33^P] ATP (1 μCi). The phosphorylation reaction was stopped by adding 5× SDS-sample buffer to the reaction mixtures before SDS-PAGE analysis. Gels were then dried and exposed to autoradiography. When muropeptides were tested, increasing amounts (from 0 to 250 µM) of TCT were added in the reaction mixture containing 2.5 μg of the PrkC or PrkCc protein, 10 mM HEPES, pH 8.0, 1 mM MgCl_2_, 2 μg of MBP and 1 mM [γ-^33^P] ATP (1 μCi).

### Limited proteolysis

For each 20 μl sample, 3 μg of PrkC or PrkCc proteins were pre-incubated for 10 min at 37 °C with 40 mM NaCl, 1 mM MgCl_2_, 10 mM Tris-HCl, pH 8.0 in the absence or presence of increasing amounts of TCT muropeptide (50, 100, 150 and 250 µM). After addition of 0.01 μg of trypsin (Promega), the reaction mixture was incubated for 10 min at 37 °C. The digestion was stopped by adding an equal volume of electrophoresis loading buffer to the assay mixtures and by heating 5 min at 100 °C before applying the samples onto a 12.5% or 15% SDS-PAGE. The gels were colored with Coomassie blue then scanned.

### Data and materials availability

Data and materials will be made available upon request.

## Electronic supplementary material

Supplemental Data
