# Supplementary material for: Dual regulation of activity and intracellular localization of the PASTA kinase PrkC during Bacillus subtilis growth
Source: Sci Rep. 2018 Jan 26;8:1660. doi: 10.1038/s41598-018-20145-2 (PMC5786024; doi:10.1038/s41598-018-20145-2)

# Dual regulation of activity and intracellular localization

# of the PASTA kinase PrkC during *Bacillus subtilis* growth.

# Frédérique Pompeo, ****Deborah Byrne,**** Dominique Mengin-Lecreulx and Anne Galinier

Supplemental Figures:


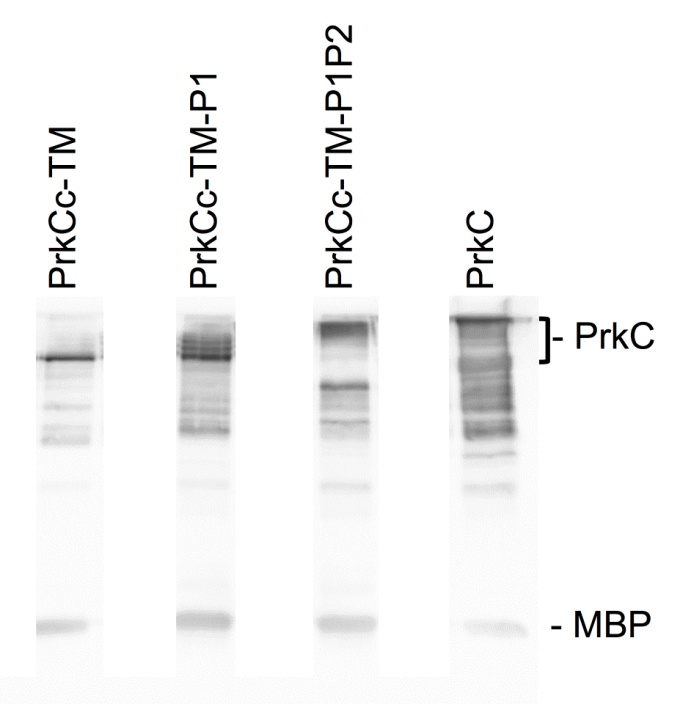


**Figure S1.** Kinase activity of full-length and truncated PrkC *in vitro.*

*In vitro* phosphorylation assays for PrkC or the truncated forms of PrkC. The recombinant purified PrkC proteins were incubated with 1 mM ATP and 2 µg of MBP at 37°C during 15 min. The tests were realized in the presence of 1 µM of each protein: PrkCc-TM, PrkCc-TM-P1, PrkCc-TM-P1P2, or PrkC. Samples were separated by SDS-PAGE and transferred to nitrocellulose membrane by electroblotting. Phosphorylated proteins were revealed with anti-P-Thr antibodies.

**
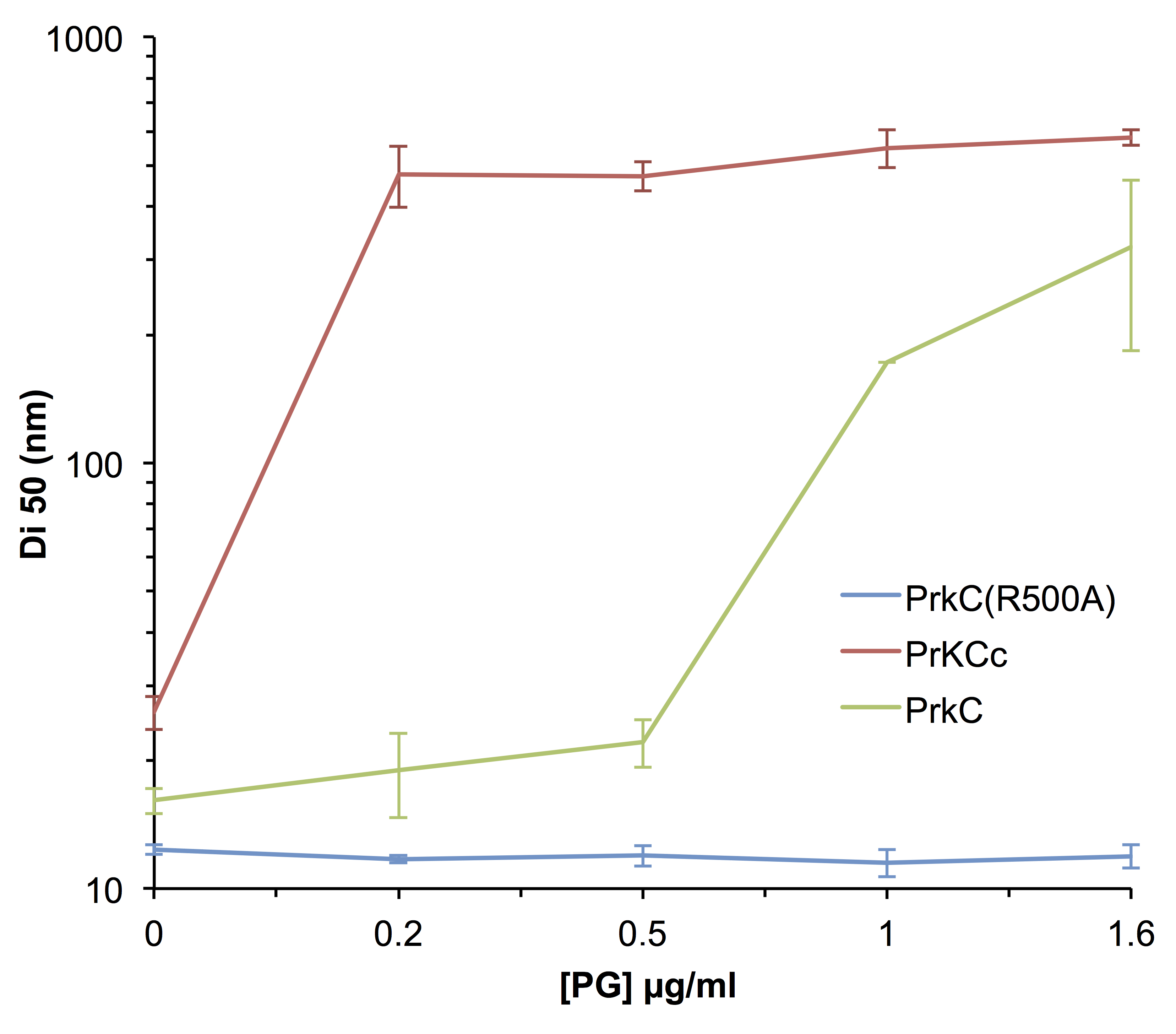
**

**Figure S2.** Binding of PG fragments to PrkC proteins.

The graph shows the diameter by intensity representing 50% of the sample (Di50) for PrkC, PrkCc and PrkC(R500A) in the presence of increasing concentrations of PG fragments from 0 to 1.6 µg/ml, calculated from DLS experiments. Di50 gradually increases suggesting an oligomerization effect of PG fragments binding on PrkC, but infers a non-specific aggregation of PrkCc with PG. Di50 remains constant for PrkC(R500A) suggesting an absence of PG fragments binding. DI50 is displayed on a logarithmic scale.

**
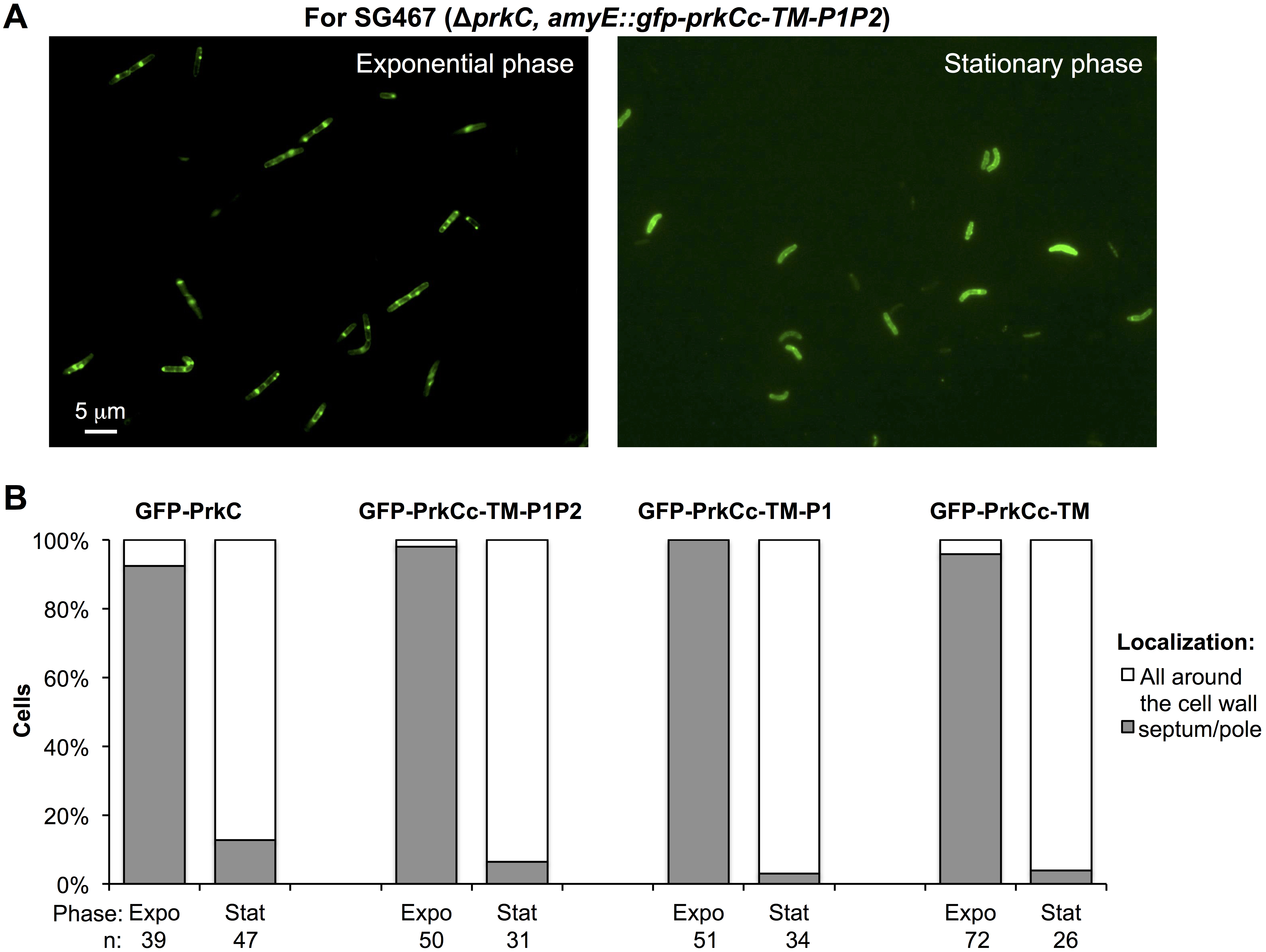
**

**Figure S3.** Localization of full-length and truncated GFP-PrkC during exponential or stationary growth phase.

Strains were grown on LB medium at 37°C and all the forms of GFP-PrkC proteins were expressed from the *P_xyl_* promoter in the presence of 0.5% xylose. PrkC localization was analyzed by fluorescent microscopy for strains SG278 (Δ*prkC*, *amyE::gfp-prkC*), SG467 (Δ*prkC*, *amyE::gfp-prkCc-TM-P1P2*), SG466 (Δ*prkC*, *amyE::gfp-prkCc-TM-P1*) and SG465 (Δ*prkC*, *amyE::gfp-prkCc-TM)* during exponential phase and after 23 hours of growth. (**A**) Cells from the strain SG467 in exponential and stationary phases are presented as example of the several possible localizations of PrkC. (**B)** The bar graph shows the percentage of cells showing each of the localizations of PrkC during growth for each strain. The number of cells analyzed per condition is mentioned below.

Supplemental Information:

**Original gels, autoradiograms and blots.**

**Parts used for the manuscript are in the red squares.**

**Figure 2**

- Gel used to construct Fig 2B:


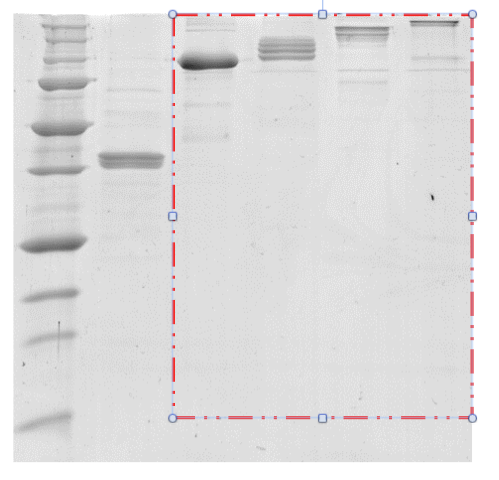


- Autoradiogram used to construct 2C:


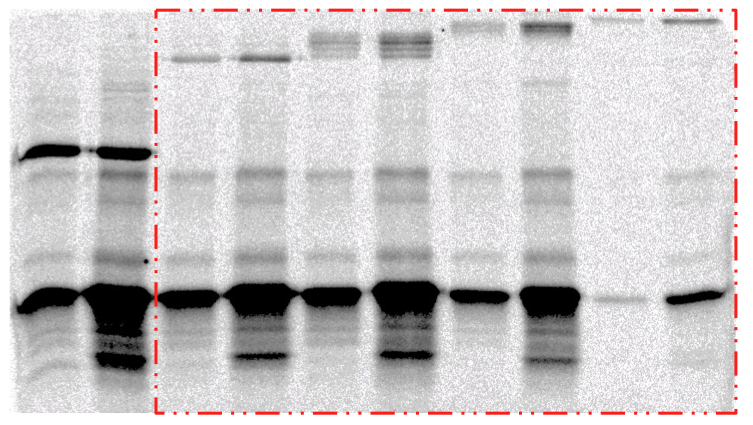


**Figure 3**

Gels used to construct Fig 3A:


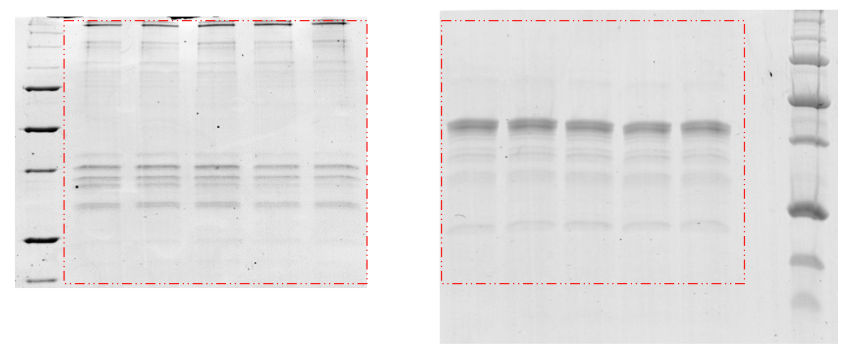


Autoradiograms used to construct Fig 3B:


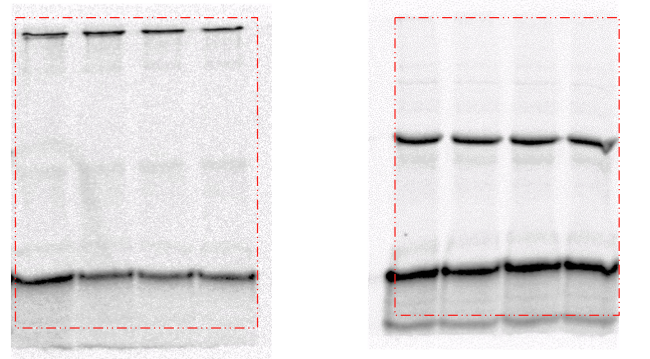


**Figure 5**

Autoradiograms used to construct Fig 5A:


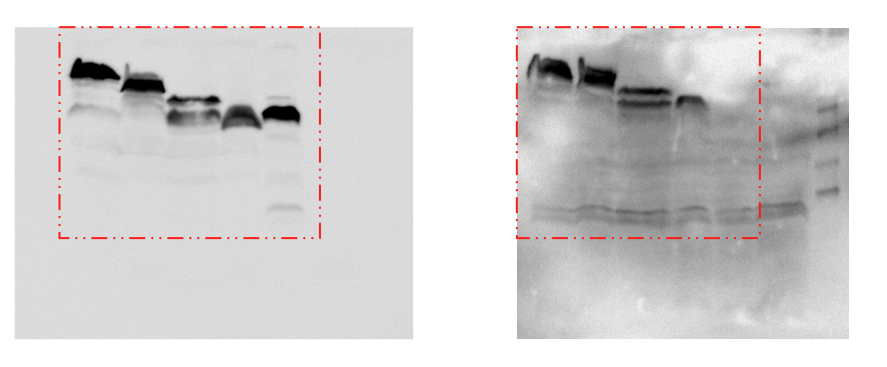


Blot used to construct Fig 5B:


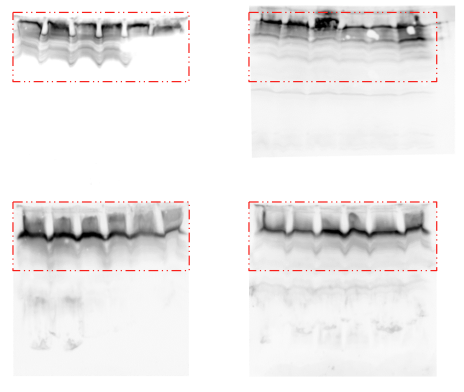


**Figure 6**

Blot used to construct Fig 6B:

**
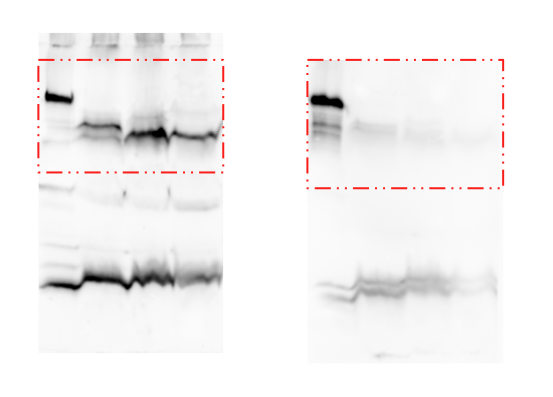
**

Blot used to construct Fig 6C:


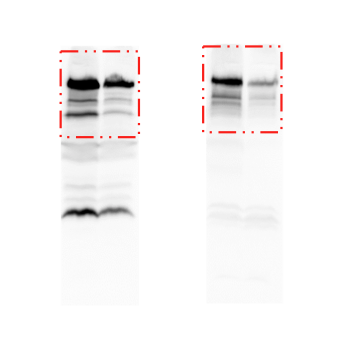


- Blot used to construct Fig. S1:


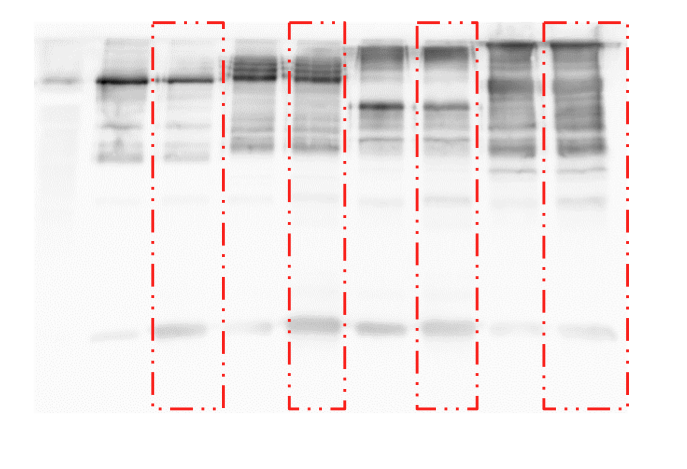

Supplement: Supplementary file 1 — Supplemental Data [file 41598_2018_20145_MOESM1_ESM.doc]
